# Association Between Platelet-to-Lymphocyte Ratio and In-hospital Mortality in Elderly Patients with Severe Trauma

**DOI:** 10.5811/westjem.61343

**Published:** 2024-01-04

**Authors:** Ji Ho Lee, Dong Hun Lee, Byung Kook Lee

**Affiliations:** * Chonnam National University Hospital, Department of Emergency Medicine, Gwangju, Republic of Korea; † Chonnam National University Medical School, Department of Emergency Medicine, Gwangju, Republic of Korea

## Abstract

**Introduction:**

The platelet-to-lymphocyte ratio (PLR) is associated with the inflammatory response in various diseases. However, studies on the use of the PLR for the prognosis of elderly patients with severe trauma are lacking. In this study, we examined the relationship between the PLR and in-hospital mortality in elderly patients with severe trauma.

**Methods:**

This retrospective observational study included elderly (≥65 years) patients who were admitted for severe trauma (as defined by an Injury Severity Score [ISS] ≥ 16) between January–December 2022. We conducted multivariate analysis to assess the association between the PLR and in-hospital mortality using logistic regression of relevant covariates. We also performed receiver operating characteristic curve analysis to examine the prognostic performance of the PLR for in-hospital mortality.

**Results:**

Among the 222 patients included in the study, the in-hospital mortality rate was 19.4% (43). The PLR of non-survivors was lower than that of survivors (62.1 vs 124.5). The areas under the curve (AUC) of the Glasgow Coma Scale (GCS) score ≤12, ISS, hemoglobin level, and PLR for predicting in-hospital mortality were 0.730 (95% confidence interval [CI] 0.667–0.787), 0.771 (95% CI 0.710–0.824), 0.657 (95% CI 0.591–0.719), and 0.730 (95% CI 0.667–0.788), respectively. The AUC of the PLR was not significantly different from that of GCS score ≤12 and ISS for predicting in-hospital mortality. Multivariate analysis showed that the PLR was independently associated with in-hospital mortality (odds ratio: 0.993; 95% CI 0.987–0.999).

**Conclusion:**

Low platelet-to-lymphocyte ratio is independently associated with in-hospital mortality in elderly patients with severe trauma.

Population Health Research Capsule What do we already know about this issue?

*Platelet to lymphocyte ratio (PLR) is part of complete blood count and is routinely implemented. Higher PLR is associated with poor prognosis of inflammatory conditions such as sepsis and intracranial hemorrhage.*

What was the research question?

*Could PLR help predict the outcome of elderly emergency department patients with severe trauma who visited the emergency department?*

What was the major finding of the study?

*a lower absolute PLR (in cells × 10^9^/L) was independently associated with in-hospital mortality (odds raito:0.993; 95% CI, 0.987–0.999). For survivors, PLR was 124.5, while those who died had PLR of 62.1.*

How does this improve population health?

*PLR alone could be helpful in predicting mortality of patients with severe trauma, but when combined with other factors, PLR can be more helpful in determining treatment direction.*

## INTRODUCTION

Trauma is a leading cause of trauma-related death and disability worldwide. Indeed, a global burden of disease study showed that in 2019 8% of all deaths worldwide were due to injury.^1^ Moreover, in 2019 109.7 million people were injured and 458,669 people died from injuries in all European countries.^2^ Trauma also has a large economic burden resulting from hospitalization, time off work, and disability. In one study, the elderly showed worse outcomes, including mortality, hospitalization rate, hemodynamic instability criteria, and anatomical and biochemical parameters.^3^ Another study showed higher mortality, longer hospital stays, and more severe complications in elderly patients with trauma than in younger patients with trauma.^4^ Therefore, it is important to rapidly identify factors that can determine prognosis in elderly patients with trauma and to provide intensive treatment when a poor prognosis is predicted.

Many triage tools for trauma have been developed, and several studies have been conducted on the effectiveness of these tools in predicting patient outcome. Clinical instruments, such as the National Early Warning Score, Modified Early Warning Score), and Acute Physiology and Chronic Health Assessment (APACHE II) score, used in critically ill patients can help predict prognosis in patients with trauma.^5^ ^,^ ^6^ Additionally, the Injury Severity Score (ISS), Revised Trauma Score, and Trauma and Injury Severity Score are commonly used tools in trauma.^7^ ^–^ ^9^ However, these tools often have cumbersome evaluation processes and subjective assessments; therefore, easier and more objective prognostic predictors should be considered.

The platelet-to-lymphocyte ratio (PLR) has good generalizability and can be calculated and obtained from routine laboratory tests at admission without further inconveniencing the patient. Studies have shown that the PLR is associated with the inflammatory response, with a higher PLR indicating poorer prognosis for patients with chronic obstructive pulmonary disease, myocardial infarction, and sepsis.^10^ ^–^ ^12^ Moreover, a previous study showed that the PLR was associated with the neurologic outcome in intracranial hemorrhage.^13^ As the PLR is routinely measured in clinical laboratories as a component of the complete blood count (CBC) and is available to most patients, it can be very useful for risk stratification in clinical decision-making. Therefore, the PLR would help to predict the outcome of elderly patients with severe trauma who visited the emergency department (ED). We examined the relationship between the PLR and in-hospital mortality in elderly patients with severe trauma.

## METHODS

### Study Design and Population

This was a retrospective observational study of elderly (≥65 years) patients with severe trauma (ISS ≥ 16) who visited the ED of Chonnam National University Hospital, a tertiary referral center in Gwangju, Korea, between January–December 2022. We collected and reviewed data from a prospectively collected trauma database at the hospital, which was nominated as a regional trauma center in South Korea in 2013. It corresponds to a Level I trauma center in the United States. Our 1,800-bed teaching hospital serves a population of three million people; and more than 500 patients with major trauma ((ISS >15) are admitted annually. Korea’s regional trauma center consists of specialists in neurosurgery, thoracic surgery, trauma surgery, orthopedic surgery, and emergency medicine; ISS is evaluated by specialists in each department. The ISS is confirmed by each specialist of the trauma care center and mutually agreed upon in case of a conflict regarding the ISS determined through regular meetings. In addition, severe trauma cases are randomly selected every year and evaluated for ISS decisions by specialists in other hospitals.

The following exclusion criteria were applied: cardiac arrest following trauma before ED visit; and missing data. This paper complies with the STROBE guidelines for reporting observational studies (Appendix 1). The institutional review board of our hospital approved the study and waived the requirement for informed consent due to the retrospective nature of the study.

### Data Collection

We obtained data on the following variables for each patient: age; gender; mechanism of trauma; systolic blood pressure (SBP, millimeters mercury); respiratory rate; pulse rate on ED arrival; initial Glasgow Coma Scale (GCS) score on ED arrival; laboratory results on arrival at the ED (CBC parameters, blood urea nitrogen [BUN], serum creatinine, and serum electrolytes); and in-hospital mortality. We calculated the PLR based on the lymphocyte and platelet counts of CBC parameters. The values of the abbreviated injury scale and ISS were evaluated based on the data from the patients’ electronic health records. The primary outcome was in-hospital mortality.

### Statistical Analysis

Continuous variables that did not satisfy the normality test are presented as median values with interquartile ranges. Categorical variables are presented as frequencies and percentages. We assessed differences between the two groups using the Mann-Whitney U-test for continuous variables. The Fisher exact test or chi-square test was used to compare categorical variables, as appropriate. Furthermore, we conducted a multivariate analysis using logistic regression of relevant covariates to predict in-hospital mortality. Variables with *P*-values <0.20 in the univariate analysis were included in the multivariate regression model. We used a backward stepwise approach and sequentially eliminated variables with *P*-values >0.10 to build a final adjusted regression model.

The results of logistic regression analysis are presented as odd ratios (OR) and 95% confidence intervals (CI). Receiver operating characteristic curve (ROC) analysis was performed to examine the prognostic performance of GCS score ≤12, ISS, hemoglobin level, and PLR for in-hospital mortality. Comparison of dependent ROC curves was performed using the DeLong method.^14^ We performed all analyses using PASW/SPSS software, version 18 (IBM Inc., Chicago, IL) and MedCalc version 19.0 (MedCalc Software, bvba, Ostend, Belgium). A two-sided significance level of 0.05 was defined as a statistically significant value.

## RESULTS

### Patient Selection and Characteristics

**Figure 1.**
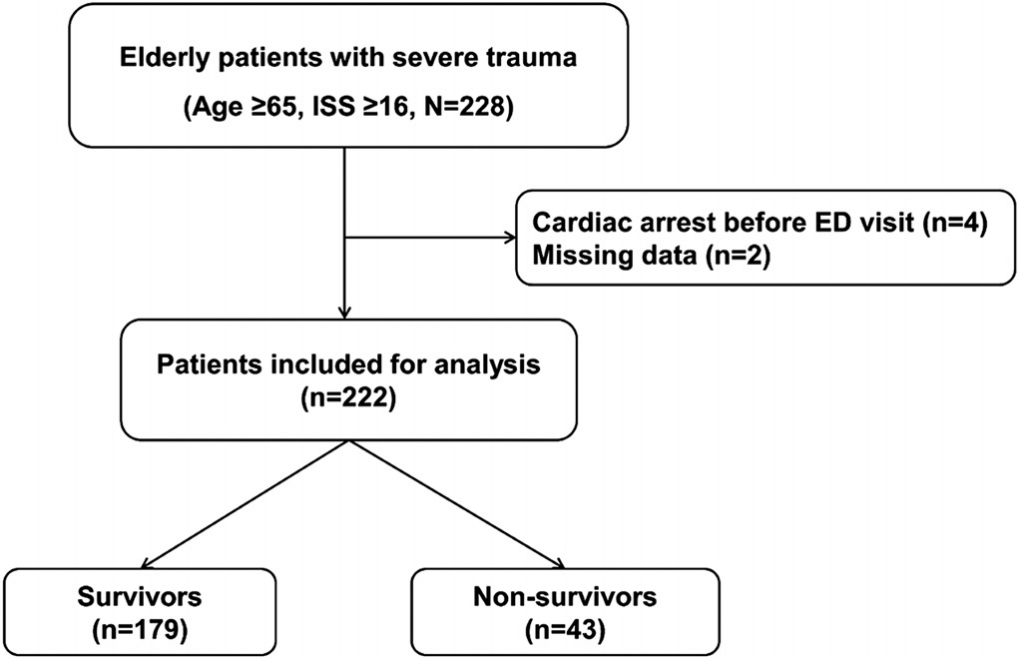
Schematic diagram showing the number of elderly patients with trauma included in the study. *ISS*, Injury Severity Score; *ED*, emergency department.

In total, 228 elderly patients with severe trauma met the inclusion criteria during the study period. After excluding patients based on the exclusion criteria, 222 patients were included in the study (Figure 1), comprising 151 men (68.0%), with a median age of 75.0 (70.0–80.8) years and an in-hospital mortality rate of 19.4% (43).

### Prognostic Performance of the ISS, GCS <12, Hemoglobin Level, and PLR for Predicting In-hospital Mortality

**Figure 2.**
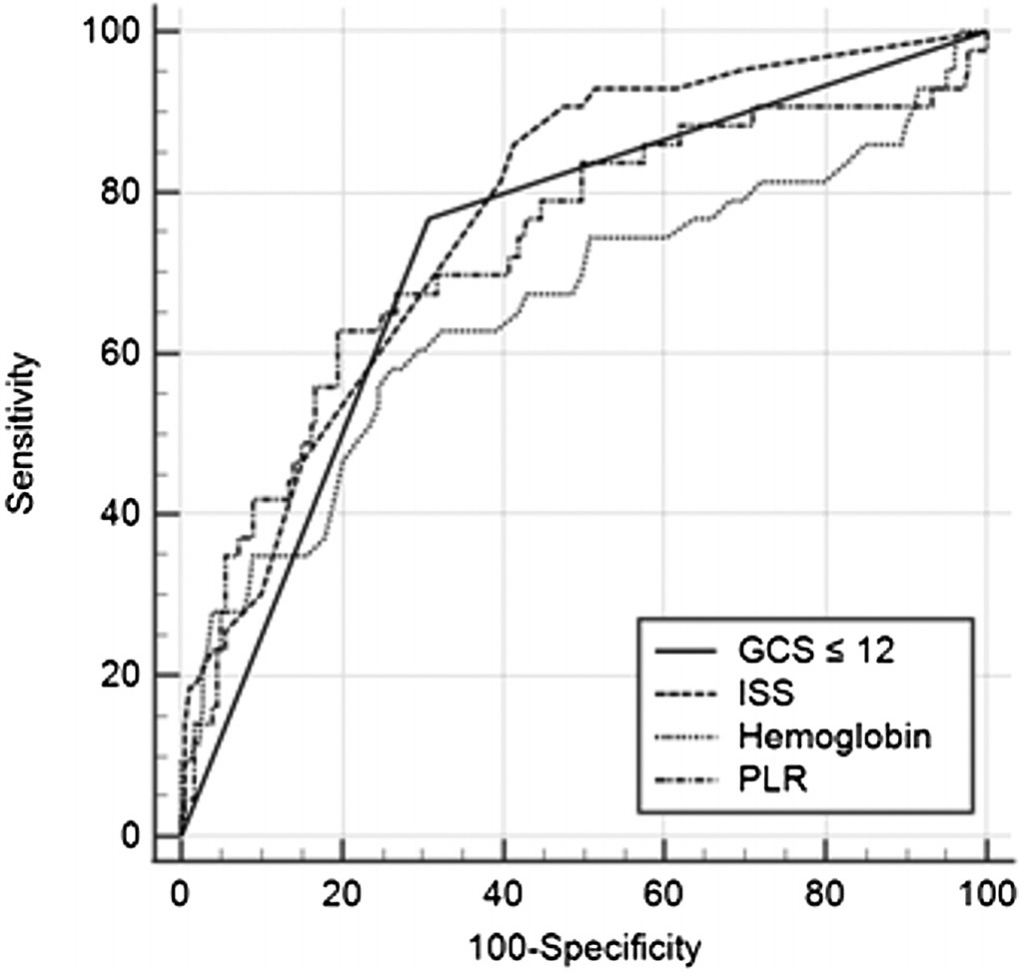
Graph showing the areas under the curves of Glasgow Coma Scale score ≤12, Injury Severity Score, hemoglobin level, and platelet-to-lymphocyte ratio for predicting in-hospital mortality. *GCS*, Glasgow Coma Scale; *ISS*, Injury Severity Score; *PLR*, platelet-to-lymphocyte ratio.

The area under the curve (AUC) of GCS score ≤12, ISS, hemoglobin level, and PLR for predicting in-hospital mortality were 0.730 (95% confidence interval [CI] 0.667–0.787), 0.771 (95% CI 0.710–0.824), 0.657 (95% CI: 0.591–0.719), and 0.730 (95% CI 0.667–0.788), respectively. The AUC of the PLR was not significantly different from that of GCS score ≤12 and ISS for predicting in-hospital mortality (Figure 2).

### Comparison of the Baseline and Clinical Characteristics Between Survivors and Non-Survivors

Table 1 shows the baseline and clinical characteristics of survivors and non-survivors. According to hospital data, non-survivors had a greater proportion of GCS scores ≤12, lower SBP, hemoglobin level, monocyte count, platelet count, and PLR, and higher ISS, lymphocyte count, red cell distribution width, and creatinine level than survivors.

**Table 1.** Comparison of the baseline characteristics of elderly patients with severe trauma according to in-hospital mortality.

Variables	Total patients (n = 222)	Survivors (n = 179)	Non-survivors (n = 43)	*P*-value
Age, years	75.0 (70.0–80.0)	74.0 (68.0–80.0)	76.0 (71.0–80.0)	0.159
Male, n (%)	151 (68.0)	120 (67.0)	31 (72.1)	0.648
Type, n (%)				1.000
Blunt	218 (98.2)	176 (98.3)	42 (97.7)	
Penetrating	4 (1.8)	3 (1.7)	1 (2.3)	
GCS score ≤12, n (%)	88 (39.6)	55 (30.7)	33 (76.7)	<0.001
Systolic blood pressure, mm Hg	130 (100–150)	130 (110–152)	90 (60–150)	<0.001
Respiratory rate, /min	20 (20–20)	20 (20–20)	20 (20–20)	0.816
Pulse rate, /min	84 (70–99)	85 (72–99)	78 (63–100)	0.140
Injury Severity Score	23 (16–25)	20 (16–25)	25 (25–30)	<0.001
Blood cell count				
White blood cell count, ×10^9^/L	12.3 (9.1–15.8)	12.3 (9.1–15.9)	12.6 (9.0–15.8)	0.808
Hemoglobin, g/dL	12.0 (10.4–13.1)	12.2 (10.9–13.2)	10.6 (8.9–12.6)	<0.001
Neutrophil count, ×10^9^/L	9.8 (6.3–12.8)	9.8 (6.3–13.0)	9.7 (6.8–12.6)	0.748
Lymphocyte count, ×10^9^/L	1.6 (1.0–2.8)	1.4 (9.7–2.4)	2.6 (1.6–3.5)	<0.001
Monocyte count, ×10^9^/L	0.7 (0.4–0.9)	0.7 (0.5–0.9)	0.5 (0.4–0.8)	0.031
Platelet count, ×10^9^/L	183 (143–226)	194 (151–241)	153 (111–191)	<0.001
PLR	107.0 (66.2–181.0)	124.5 (75.7–204.6)	62.1 (36.2–104.8)	<0.001
Red cell distribution width, %	13.0 (12.4–13.7)	12.9 (12.4–13.6)	13.4 (12.6–13.8)	0.051
Kidney function				
Blood urea nitrogen, mg/dL	17.4 (13.8–21.3)	17.4 (13.9–21.4)	16.5 (13.4–21.1)	0.647
Creatinine, mg/dL	0.8 (0.7–1.0)	0.8 (0.6–1.0)	0.9 (0.8–1.1)	0.036
Serum electrolytes				
Sodium, mmol/L	139 (137–141)	139 (137–141)	139 (137–142)	0.530
Potassium, mmol/L	4.0 (3.6–4.3)	4.0 (3.6–4.2)	3.9 (3.6–4.3)	0.554
Chloride, mmol/L	105 (102–107)	105 (102–107)	106 (103–108)	0.078

*GCS*, Glasgow Coma Scale; *PLR*, platelet-to-lymphocyte ratio; *mm Hg*, millimeters of mercury; *g/dL*, grams per deciliter; *L*, liter; *mg*, milligrams; *mmol*, millimole.

### Multivariate Analysis Using Logistic Regression for Predicting In-hospital Mortality

Table 2 shows the results of the multivariate analysis for predicting in-hospital mortality. After adjusting for confounders, GCS score ≤12 (OR 4.317, 95% CI 1.830–10.181), ISS (OR 1.103, 95% CI 1.033–1.177), hemoglobin level (OR 0.753, 95% CI 0.608–0.931), and PLR (OR 0.993, 95% CI 0.987–0.999) were independently associated with in-hospital mortality.

**Table 2.** Multivariate logistic regression analysis for predicting in-hospital mortality in elderly patients with severe trauma.

	Adjusted OR (95% CI)	*P*-value
Age, years	1.026 (0.966–1.089)	0.410
GCS score ≤12	4.317 (1.830–10.181)	<0.001
Systolic blood pressure, mm Hg	0.994 (0.984–1.004)	0.260
Pulse rate, /min	0.984 (0.964–1.004)	0.111
Injury Severity Score	1.103 (1.033–1.177)	0.003
Hemoglobin, g/dL	0.753 (0.608–0.931)	0.009
Monocyte count, ×10^9^/L	0.999 (0.998–1.000)	0.152
PLR	0.993 (0.987–0.999)	0.017
Red cell distribution width, %	1.153 (0.883–1.504)	0.295
Creatinine, mg/dL	0.671 (0.331–1.358)	0.267
Chloride, mmol/L	0.961 (0.879–1.050)	0.376

*OR*, odds ratio; *CI*, confidence interval; *GCS*, Glasgow Coma Scale; *PLR*, platelet-to-lymphocyte ratio; *mm Hg*, millimeters of mercury; *min*, minute; *g/dL*, grams per deciliter; *L*, liter; *mg*, milligram; *mmol*, millimole.

## DISCUSSION

In this retrospective observational study, the PLRs of non-survivors were lower than those of survivors in elderly patients with severe trauma. Additionally, the PLR showed similar predictive power to the GCS score and ISS for in-hospital mortality in elderly patients with severe trauma upon ED arrival. Lymphocyte and platelet counts of non-survivors were significantly different from those of survivors. Platelet activation results in endothelial damage and promotes neutrophil extracellular traps and microthrombus formation.^15^ ^,^ ^16^ Several studies have reported that low platelet counts are related to multiorgan dysfunction syndrome in patients with trauma.^17^ ^,^ ^18^ Platelets induce the secretion of inflammatory cytokines, which interact with neutrophils, T cells, and macrophages.^17^ ^,^ ^18^ These platelet-induced complex inflammatory responses may contribute to in-hospital mortality in patients with trauma.

Several studies have reported that platelet function declines with age in elderly patients and that this relationship is associated with prognosis.^19^ ^–^ ^21^ Lymphocytes, including T cells, B cells, and natural killer cells, are the major cellular component of the humoral and cell-mediated immune system.^22^ Acute lymphocytosis in the early stages of trauma is related to the degree of injury and mortality.^23^ In elderly patients, a high lymphocyte count has been shown to be associated with nutritional status or sepsis associated with delirium.^24^ ^,^ ^25^ Thus, in the present study the effect of lymphocytes and platelets, which are related to prognosis, was further increased through the PLR in elderly patients with severe trauma. The PLR has many advantages and is widely used in the clinical field. The PLR is not only simple and easy to calculate, but the CBC test, which includes the PLR, is widely available and inexpensive, allowing it to be used in almost all EDs worldwide, including those in developing countries.

Several studies have reported that a low PLR is associated with mortality in patients with trauma, similar to the findings of the present study.^26^ ^–^ ^28^ In the study by Ke et al, the PLRs of non-survivors were higher than those reported in the present study (124.3 vs 62.1).^26^ However, in that previous study, the mean age of the included patients was <65 years, and there was also a proportion of patients with an ISS <16.^26^ High PLRs have also been shown to be associated with prognosis in non-traumatic medical problems, including tumors, sepsis, and heart failure.^29^ ^,^ ^30^ In the early stages of trauma, the response of various inflammatory or coagulation systems may be different from that of other diseases. Further studies are needed to clarify the relationship between PLR and disease or trauma.

Our results showed that a low hemoglobin level was associated with in-hospital mortality in elderly patients with severe trauma. The results of a previous study suggested that in patients with severe trauma without prehospital intravenous fluid administration, decreased hemoglobin levels on arrival may be associated with the severity of trauma and the need for hemostasis.^31^ Another study reported that a low hemoglobin level was correlated with poor neurologic outcomes in patients with traumatic brain injury.^32^ Low hemoglobin levels can be proportional to primary volume loss and result in secondary brain damage due to cerebral hypoxia.^32^

The GCS is a routine component of neurological examination for critically ill patients with trauma. Many studies have shown that a low GCS score is associated with poor prognosis in elderly patients with trauma, as shown in the present study.^33^ ^,^ ^34^ However, it is difficult to predict prognosis with the GCS because it does not involve brainstem reflexes, nor does it accurately describe the verbal status of intubated patients. In particular, in elderly patients with trauma, the measurement of the GCS motor response can be inaccurate, requiring more careful measurement.^35^

Several studies have reported that a high ISS value is associated with mortality in elderly patients with trauma.^36^ ^,^ ^37^ Indeed, one such study reported the predictive power of ISS for 30-day mortality as 0.66 (95% CI 0.59–0.74), which is lower than that reported in our study, as well as higher ISS values and mortality than in our study.^37^ Additionally, as one of our inclusion criteria was patients with an ISS of ≥16, the relationship between ISS and mortality was more pronounced.

Elderly patients have more frequent loss of consciousness than non-elderly patient; this is often due to various metabolic causes as well as structural problems in the head. Thus, GCS score may be difficult to measure and less accurate in elderly patients than in non-elderly patients.^38^ And GCS measurement is affected by sedatives or neuromuscular blockade, whereas PLR obtained by simple calculation through CBC can provide more objective information about patients than GCS measurement. In addition, PLR does not require imaging studies or related specialists, who are needed to determine ISS. However, in this study, PLR showed similar AUC values compared to GCS for predicting in-hospital mortality, which was not superior. The PLR alone cannot be a predictor of mortality; however, in combination with other factors, the PLR can be a warning sign or determine the direction of treatment.

## LIMITATIONS

This study has several limitations that warrant discussion. First, it was a retrospective study performed at a single center; thus, our findings are not immediately generalizable to the overall population. Additional multicenter studies with larger samples and prospective designs are necessary to substantiate our findings. Second, other inflammatory markers, such as cytokines and chemokines, were not investigated in this study. In particular, studies on the relationship between the lymphocyte subgroup and elderly trauma are needed. Third, as we investigated the relationship between the PLR at ED visit and prognosis, it is necessary to investigate the relationship between serial PLR and prognosis in elderly patients with trauma. Finally, because we are a small group, we played multiple roles of study designer, case identifier, data abstractor, data analyst, and author. There are limitations in blinding and monitoring because small groups carry out these roles by themselves. However, efforts were made to address the bias that could occur with a retrospective observational study and, fortunately, our hospital is constructing a dataset under the operation of a regional trauma center when a severely ill patient visits the hospital.

## CONCLUSION

Low platelet-to-lymphocyte ratio is independently associated with in-hospital mortality in elderly patients with severe trauma. The association remained significant after adjustment for hospital risk factors and important laboratory variables.

## Supplementary Information




